# Quantitative Assessment of Hyperpigmentation Changes in Human Skin after Microneedle Mesotherapy Using the Gray-Level Co-Occurrence Matrix (GLCM) Method

**DOI:** 10.3390/jcm12165249

**Published:** 2023-08-11

**Authors:** Iga Wawrzyk-Bochenek, Mansur Rahnama, Sławomir Wilczyński, Anna Wawrzyk

**Affiliations:** 1 Department of Basic Biomedical Science, Faculty of Pharmaceutical Sciences in Sosnowiec, Medical University of Silesia, Kasztanowa 3, 41-205 Sosnowiec, Poland; iga.wawrzyk97@gmail.com (I.W.-B.); swilczynski@sum.edu.pl (S.W.); 2 Chair and Department of Oral Surgery, Medical University of Lublin, Chodźki 6, 20-093 Lublin, Poland; rahnama.m@interia.pl; 3 Silesian Park of Medical Technology Kardio-Med Silesia in Zabrze, M. Curie Skłodowskiej 10C, 41-800 Zabrze, Poland

**Keywords:** skin hyperpigmentation, microneedle mesotherapy, GLCM

## Abstract

Aim: The aim of the study was to quantitatively assess the effectiveness of microneedle mesotherapy in reducing skin discoloration. The results were analyzed using the gray-level co-occurrence matrix (GLCM) method. Material and methods: The skin of the forearm (7 × 7 cm) of 12 women aged 29 to 68 was examined. Microneedle mesotherapy was performed using a dermapen with a preparation containing 12% ascorbic acid. Each of the volunteers underwent a series of four microneedle mesotherapy treatments. The effectiveness of the treatment was quantified using the methods of image analysis and processing. A series of clinical images were taken in cross-polarized light before and after a series of cosmetic procedures. Then, the treated areas were analyzed by determining the parameters of the gray-level co-occurrence matrix (GLCM) algorithm: contrast and homogeneity. Results: During image pre-processing, the volunteers’ clinical images were separated into red (R), green (G) and blue (B) channels. The photos taken after the procedure show an increase in skin brightness compared to the photos taken before the procedure. The average increase in skin brightness after the treatment was 10.6%, the average decrease in GLCM contrast was 10.7%, and the average homogeneity increased by 14.5%. Based on the analysis, the greatest differences in the GLCM contrast were observed during tests performed in the B channel of the RGB scale. With a decrease in GLCM contrast, an increase in postoperative homogeneity of 0.1 was noted, which is 14.5%.

## 1. Introduction

Skin discolorations in the form of hypopigmentation or hyperpigmentation are caused by uneven accumulation of pigment due to quantitative or qualitative deficiencies in the transfer, synthesis and degradation of melanin. Discoloration can be congenital or result from mechanical damage, hormonal changes, inflammation, too long exposure to UV radiation or improper care [1,2].

One of the methods used to eliminate hyperpigmentation changes is microneedle mesotherapy consisting of micropuncture of the skin. Microneedling is a minimally invasive and safe medical procedure [3,4,5,6].

It has long been used for therapeutic purposes or as a method of administering drugs in dermatological diseases [7]. Stimulations and processes occurring in the skin after microneedling can lead to biological changes and support the treatment of skin diseases [8,9,10,11].

When performing micropunctures, microtrauma and microbleeding occur. Blood leaks from damaged blood vessels, which activates platelets. Micropunctures stimulate fibroblasts responsible for the production of collagen and elastin. The increase in collagen and elastin in the skin can reach 400% after 6 months, while the increase in the thickness of the granular layer takes up to a year [5,12,13,14].

The process of skin stimulation by microneedle mesotherapy treatment is divided into three stages: initiation of inflammation, formation of new collagen and elastin fibers, as well as new vessels and tissues [14].

The high effectiveness of microneedle mesotherapy is noted in the treatment of discoloration reduction, where during the treatment, due to the created microchannels, an appropriate active substance is delivered, whose task is to lighten discolorations and inhibit tyrosinase. During the treatment, skin repair processes are initiated, due to which the proper production of melanocytes in the area covered by discoloration is stimulated [15].

Microneedle mesotherapy is performed using a dermapen (an automatic device with adjustable needle length up to 3 mm and various operating modes depending on the type of skin surface), a dermo-roller (rolls with 192 microneedles with a diameter of 0.1 mm and an average length of 1.5 mm) and derma-stamp (a miniature version of the dermo-roller) [16,17].

The selected depth of micropunctures during the procedure depends on the area of the skin, the thickness of the epidermis and individual characteristics. Typically, the optimal depth is determined by observing punctate bleeding [3,4].

Needles are usually inserted into the skin of the face to a depth of 0.3 to 0.5 mm. It is estimated that this depth on the skin of the neck and cleavage ranges from 1.5 to 3 mm [4].

The effectiveness of the treatment can be increased by adding active substances [14]. The active substances penetrate the deeper layers of the skin immediately after the puncture or up to three days after the procedure [3,15].

The active substances used in the microneedle mesotherapy treatment are selected individually depending on the needs and condition of the skin and the intended effects. Mesotherapy commonly uses ingredients naturally found in the skin, e.g., platelet-rich plasma, vitamins A, B, C, E and K, hyaluronic acid, minerals and nucleic acids [18].

Microneedle mesotherapy with vitamin C effectively improves skin tone [19].

Vitamin C has been proven to improve pigmentation after microneedling [20].

The effects after the treatments are assessed subjectively by the researcher. Few results of histopathology, ultrasonography and electron microscopy have been published for a more professional assessment of the procedure [21].

In cosmetology, the effects of microneedle mesotherapy are mainly assessed qualitatively based on clinical images [22].

The skin photos taken before and after the procedures ensure reproducibility and standardization of the procedures [23].

Photographs in dermatology and cosmetology include facial features, landmarks, and also many other clinically relevant details such as pigmentation, skin texture and pore size, which are evaluated especially when determining the effectiveness of facial resurfacing procedures [24].

Along with the development of cosmetology, in order to obtain more accurate results and a more detailed analysis of the effectiveness of treatments, precise quantitative methods began to be used in this field. Using specialized computer software enables comparison and quantitative presentation of the results collected during the documentation of individual stages of therapy. These methods allow for a detailed interpretation of the obtained results and the possibility of further improvement of individual treatment procedures [25].

GLCM (gray-level co-occurrence matrix) analysis, i.e., the so-called gray-level coexistence matrix, is a statistical method of data analysis that examines the relationship between the neighborhood of pixels in the examined images. This method uses pixel-to-pixel correlation and typically targets pixels that are close together. It is determined by the angle and the distance between pixels. In the GLCM method, the analysis of neighborhood relations consists of creating a matrix whose elemental values reflect the number of occurrences of the pairs of pixels with the same colors at a given distance between them, in a given area and in a given direction for the entire image or its fragment. The columns in the GLCM matrix correspond to the gray-level values of the adjacent points, and the rows correspond to the gray-level values of the reference points. In the analyzed area, the values of the matrix elements determine how many times a pixel of a given shade was adjacent to a pixel of a different shade. On the basis of the neighborhood matrix, individual statistical features of the analyzed area are determined [25].

Due to the use of the GLCM method, it is possible to precisely determine the degree of reduction in a given skin defect and to note which parameters used during the treatment brought the greatest effect. This allows for the development of newer treatment procedures and the documentation of their effects.

## 2. Materials and Methods

### 2.1. Patients

Twelve women between the ages of 29 and 68 participated in the study. All patients consented to the procedure and measurements. All volunteers had skin phototype II or III and heterogeneous skin color resulting from the presence of post-sun lesions. Each of the subjects underwent a series of 4 microneedle mesotherapy treatments performed every 14 days. Contraindications such as the presence of skin lesions (except sun changes) at the site of the procedure, cancer, pregnancy, breast-feeding and blood clotting disorders were excluded in the patients. 

### 2.2. Microneedling Technique

The treatments were performed using the ULTIMA M7-C, Dr Pen dermapen, which was used perpendicularly at a depth of 1 mm into the skin to obtain satisfactory results. For detailed analyses, images corresponding to the area of interest (ROI—region of interest) were selected. The images used were taken in cross-polarized light, before the first treatment and two weeks after the fourth treatment of microneedle mesotherapy.

### 2.3. Photo Acquisition

The Fotomedicus (system from Elfo, Łódź, Poland) was used to acquire skin images. It is a device with software that allows one to take pictures with strictly defined parameters, which ensures the creation of good, repeatable medical documentation. The main element of the equipment is a reflector with a camera and a ring-type flash with constant flash energy. The flash energy is current-controlled, and the light used has a very high CRI (color rendering index) of >90%. The Fotomedicus system enables the acquisition of images in non-polarized and cross-polarized light for the polarization angle of θ = 90°. Such polarization is obtained by using two linear polarizing filters, where the first is placed on the camera lens, and the second filter on the light sources. Cross polarization of light allows electromagnetic radiation to penetrate deeper into the epidermis than in the case of unpolarized light. The image is devoid of reflections and other artifacts related to the reflection and dispersion of light, e.g., on the sebaceous coating. This allows for a much better visualization of changes in the dermis and epidermis at the expense of losing part of the information about the morphometric structure of the skin surface, which in this case was not the subject of the analysis.

Matlab Version 7.11.0.584 (R2010b) 2018 software was used to analyze the images. Dedicated software developed specifically for this application was used to quantitatively identify homogeneity and contrast parameters.

### 2.4. Preparation

The preparation was applied to an area of 7 cm × 7 cm on the forearm (region of interest). Before the treatment, the skin was prepared, i.e., cleansed with a surfactant and disinfected with 76% ethanol, and micropunctured using an ampoule containing vitamin C. Ampoules containing 12% vitamin C in the form of ascorbic acid were used. 

### 2.5. Quantitative Analysis

The quantitative assessment of the effectiveness of the microneedle mesotherapy treatment was carried out using the gray-level co-occurrence matrix method. GLCM analysis, i.e., the so-called gray-level co-occurrence matrix, is a statistical method of data analysis that examines the relationship between the neighborhood of pixels in the images under examination. This method uses pixel-to-pixel correlation and typically targets pixels that are in close proximity to each other. It is defined by the angle and the distance between pixels. In the GLCM method, the analysis of neighborhood relations consists of creating a matrix whose element values reflect the number of occurrences of pairs of pixels with the same colors at a given distance between them, in a given area and in a given direction, on the entire image or its fragment. The columns in the GLCM matrix correspond to the gray level values of the adjacent points, and the rows correspond to the gray level values of the reference points. In the analyzed area, the values of the elements in the matrix determine how many times a pixel of a particular shade was adjacent to a pixel of a different shade. On the basis of the neighborhood matrix, individual statistical features of the analyzed area are determined. In the adopted research model, images with a resolution of 800 × 727 pixels were used for the GLCM analysis.

### 2.6. The Procedure of the Applied Treatment

After a detailed consultation before the procedure and a thorough analysis of the skin and its needs, possible contraindications were excluded. One ampoule was applied to the volunteer’s skin in the ROI area and microneedling was performed immediately after its application. An area of 7 × 7 cm was marked on the skin, which was used with needles to a depth of 1.0 mm for 1.5–2 min. After the procedure, the inflammation caused by the punctures caused redness and swelling of the skin in 8 volunteers. Symptoms in all patients were accompanied by a slight burning sensation and skin tightening.

### 2.7. Statistical Analysis

GLCM analysis, i.e., the so-called gray-level co-occurrence matrix, determines how many times a pixel in an image with a given brightness is adjacent to a pixel with a different brightness. It should be noted that the vascular lesion is most visible when the contrast between the lesion and unaffected skin is greatest. Thus, the subjective perception of pigmentation changes is influenced by the number of vascular changes in a given area and their intensity (contrast). Both of these parameters are quantitatively represented in the analysis. In the GLCM analysis, pixels located in the direct line are most often taken into account. 

The GLCM functions provide an accurate, numerical characterization of an image’s texture by calculating the frequency of co-occurrence of pixel neighborhoods with operator-specified gray-level differences. In the GLCM matrix, the number of columns and rows is equal to the number of gray levels (G). Image analysis can be performed in different directions: vertically (90°), horizontally (0°) and diagonally (45 or 135°).

Before generating the matrix, one must specify the offset d, i.e., the distance between pixels, and the angle θ at which they will be analyzed. In this study, pixels in the horizontal direction (θ = 0°) located in the immediate vicinity (d = 1) were studied. In practice, this means that the matrix’s row x and column y contain information about how often a pixel with a certain level of gray (brightness) is located directly to the right of a pixel with a different, defined level of gray. This numerically identifies the element P of the matrix, which informs about the frequency with which two pixels separated by a distance (Δx, Δy) occur in a given neighborhood.

GLCM contrast and GLCM homogeneity were determined for the recorded images.

The GLCM contrast in the conducted research model is understood as follows:

$$\left(\underset{i,j}{\sum }{\left|i-j\right|}^{2}p\left(i,j\right)\right)$$

where:

*i*—brightness of the tested pixel

*j*—brightness of the neighboring pixel

A derivative of the GLCM contrast is image homogeneity. In the proposed research model, homogeneity is understood as follows:

$$\left(\underset{i,j}{\sum }\frac{p\;\left(i,j\right)}{1+\left|i-j\right|}\right)$$

where:

*i*—brightness of the tested pixel

*j*—brightness of the neighboring pixel

GLCM contrast is a measure of local variation in pixels, while homogeneity provides information about the homogeneity of pixels in the image. This means that the greater the homogeneity and the lower the contrast, the more homogeneous the skin color is (no pigmentation changes). However, the greater the contrast and less homogeneity, the more differentiated the skin tone and the more intense the pigmentation changes.

Microsoft Excel 2013 was used to analyze the obtained results. Bar charts, line charts and data tables were developed in Microsoft Excel 2013. Based on the collected results, a database was created, which was subjected to interpretation and statistical analysis. The results were used to calculate the brightness, GLCM contrast and homogeneity for each test subject, and then the average values for all patients were compiled. In the adopted research model, the brightness presented in the form of a histogram is a factor defining grayscale images in which pixel colors are described by a single number in the range of 0–255. The created histogram describes how many pixels of a given shade of gray are present in the image obtained during the tests. Thus, the higher the brightness of the image, the less visible the hyperpigmentation changes are.

## 3. Results

Clinical photography supported by dedicated image analysis and processing methods was used to qualitatively assess the effects of the procedure.

**Figure 1:**
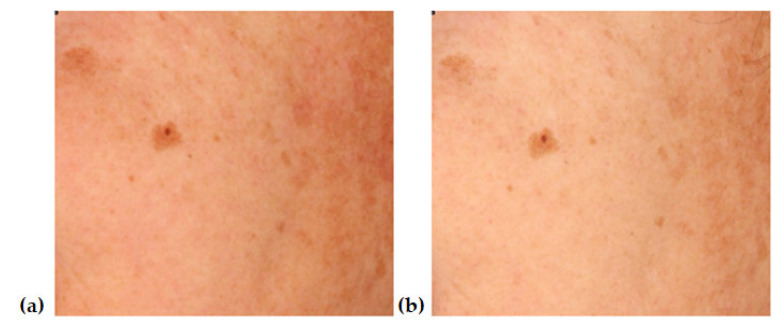
ROI image recorded in visible light in RGB space before (**a**) and after (**b**) procedure.

**Figure 2:**
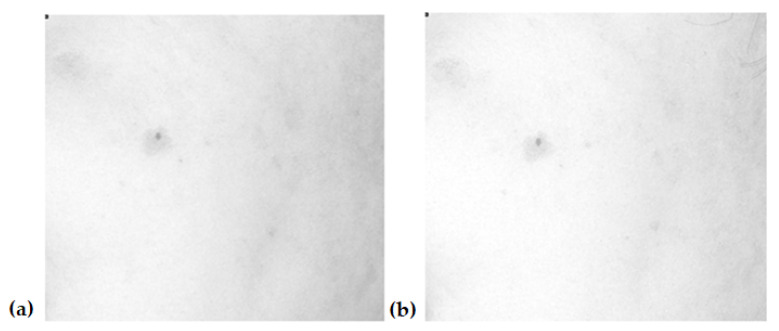
ROI image recorded in visible light—red channel (R), before (**a**) and after (**b**) procedure.

**Figure 3:**
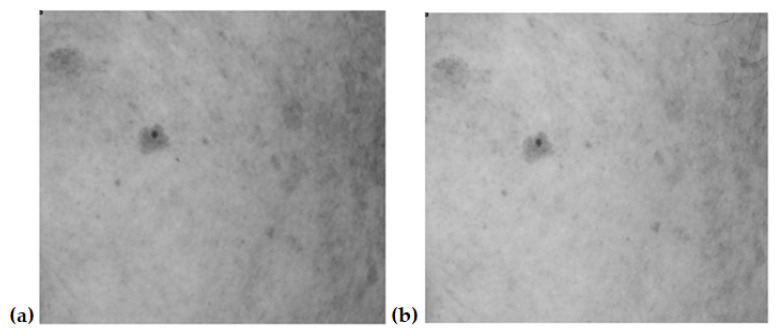
ROI image recorded in visible light—green channel (G), before (**a**) and after (**b**) procedure.

**Figure 4:**
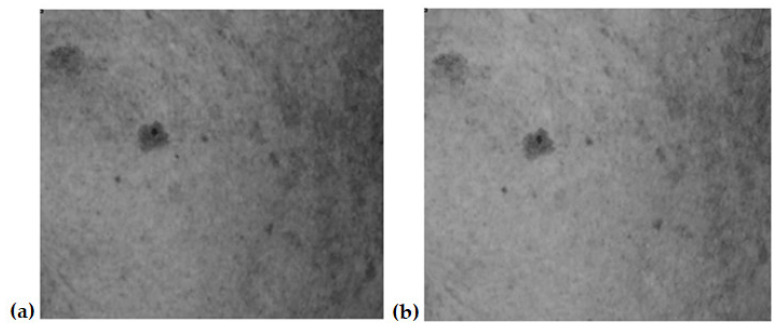
ROI image recorded in visible light—blue channel (B), before (**a**) and after (**b**) procedure.

Figure 1, Figure 2, Figure 3 and Figure 4 show an example image of Volunteer 1’s ROI taken under cross-polarized light. The photos show hyperpigmentation changes in the skin, analyzed in the RGB scale. They were made before and after a series of microneedle mesotherapy treatments.

### 3.1. Brightness Analysis

In the adopted research model, the brightness presented in the form of a histogram is a factor defining grayscale images in which pixel colors are described by a single number in the range of 0–255. The created histogram describes how many pixels of a given shade of gray are present in the image obtained during the tests. Thus, the higher the brightness of the image, the less visible the hyperpigmentation changes are. The results (Table 1) displayed in the photos taken after the procedure showed an increase in skin brightness compared to the photos taken before the procedure. The average brightness before the procedure was 156.1 ± 10.68, while after the procedure it was 174.6 ± 11.05, which was an increase of 18.5. The average increase in brightness was about 10.6%. The largest increase in brightness of about 20% was observed in patient number 11, which was 35.07, whereas the smallest increase in brightness of about 5.5% was observed in patient number 12, which was 8.74.

### 3.2. GLCM Contrast Analysis

In order to verify the sensitivity of the adopted research model to local changes in the position of the ROI area, the repeatability of the results with a slight displacement of the ROI area was verified. For this purpose, the GLCM parameters of the given ROI were calculated and then shifted by 10 mm along the long axis of the forearm and compared with the contrast and homogeneity parameters before and after the shift. Then, the ROI area was shortened by 10 degrees and the obtained GLCM parameters were also compared. In both cases, changes in GLCM parameters did not exceed 1%. This indicates the correctness of the adopted research model and the high repeatability of results.

The GLCM contrast in the adopted research model determines the difference in the gray levels of the pixels that make up the image. The GLCM contrast value shows the severity of hyperpigmentation changes in the skin. Thus, the lower the contrast value, the less visible the hyperpigmentation changes are, so the skin is more uniform. The results obtained during the GLCM contrast test (Table 2) showed statistically significant differences in the GLCM contrast between the measurement made before and after the procedure. The average GLCM contrast before the procedure was 7.5 ± 1.5, whereas after the procedure it was 6.7 ± 1.2, so we observed a decrease in the GLCM contrast by 0.8, i.e., which was about 10.7%. The greatest decrease in GLCM contrast of approximately 13.5% was observed in patient number 11, which was 1.2, whereas the smallest decrease in GLCM contrast of approximately 5.5% was observed in patient number 5, which was 0.24.

### 3.3. GLCM Homogeneity Analysis

In the adopted research model, GLCM homogeneity is described as a feature of specific image regions that are homogeneous, i.e., homogeneous in terms of gray levels. Thus, the greater the homogeneity of a given image, the less visible the hyperpigmentation changes are, so the skin is more uniform. The results obtained during the homogeneity test (Table 3) showed statistically significant differences between the measurements made before and after the treatment. The average homogeneity before the treatment was 0.62 ± 0.12, whereas after the procedure it was 0.73 ± 0.13, so we observed an increase in homogeneity by 0.11, i.e., about 15%. The largest increase in GLCM homogeneity of about 34.5% was observed in patient number 8, which was 0.3, whereas the smallest increase in homogeneity of about 4.4% was observed in patient number 1, which was 0.03.

### 3.4. RGB Scale Analysis

In the adopted research model, both changes in GLCM contrast and homogeneity were tested in the RGB scale (Table 4), in which colors are described as a mixture of three primary colors: R-red/red, G-green/green and B-blue/blue. Based on the analysis, the greatest differences in the GLCM contrast were observed during the tests performed in the B channel of the RGB scale. The average GLCM contrast values recorded in canal B before the procedure were 12.79, whereas after the procedure they decreased by 9.97, which gives a difference of 2.82—a GLCM contrast difference of 22%. With a decrease in GLCM contrast, an increase in postoperative homogeneity of 0.1 was noted, which was 14.5%.

## 4. Discussion

Improper skin tone may be caused by exogenous or endogenous pigmentation disorders [26].

Hyperpigmentation refers to a skin condition that increases pigmentation [27].

Microneedle mesotherapy is used in cosmetology and aesthetic medicine to improve skin tone. The use of microneedling in the treatment of skin lesions is the subject of many studies, while the impact of this type of treatment on pigmentation changes in the skin is poorly understood.

Despite a small number of studies, the safety and effectiveness of microneedling in the treatment of diseases associated with pigmentation disorders in the course of melasma, periorbital hypermelanosis and vitiligo have been demonstrated [7].

Microneedle mesotherapy is performed on the skin of the face, neck and cleavage, as well as on any part of the body affected by unwanted skin lesions, using needles of different lengths [28].

The effect of the procedure depends on the depth of punctures [14,28].

Needles inserted into thick and oily skin should be longer than those used for thin skin. The routinely selected needle length is 1–2 mm [29,30].

In this study, the needles were inserted to a depth of 1.0 mm since the area examined was the skin of the forearm. The length of the needles was selected based on scientific reports.

The high effectiveness of microneedle mesotherapy is noted in the treatment of discoloration reduction, where during the treatment, due to the created microchannels, an appropriate active substance is delivered, whose task is to lighten discolorations and inhibit tyrosinase [15].

Previous studies have shown that the simultaneous use of 10% trichloroacetic acid and microneedling caused skin lightening in 92% of patients with periorbital melanoma [31,32].

In 2013, the effect of microneedling on severe melasma in 60 patients was described. In the microinjection group at the end of the third follow-up visit, there was an improvement in the MASI (melasma area and security index) scale of 35.72% compared to 44.41% in the microinjection group. In 26.09% of the cases, microneedling improved skin tone by 50% on this scale [33].

It should be noted, however, that the MASI scale is semi-quantitative and the obtained results may vary depending on, among others, the type of skin illumination (color temperature of light), the angle of light incidence on the skin, the presence of other skin lesions, the experience of the doctor performing the examination and the fatigue of the doctor performing the examination. Thus, reporting differences in improvement with an accuracy of hundreds of percent is only an element resulting from statistical calculations and not the actual accuracy of estimating changes [34].

Therefore, comparing results in which the improvement is not spectacular may be difficult, and sometimes even impossible, due to the low sensitivity of the naked human eye to changes in skin color and texture. It should also be remembered that standard scales for color analysis of objects do not always work well in biomedical research. For example, the L*a*b color space is the correct choice of space from a scientific point of view [35]. However, the sensitivity of the human eye is different for different colors. Thus, a 10% change in the saturation of blue is perceived differently by the sense of sight than a 10% change in the saturation of green. Therefore, the GLCM method used in this case has an advantage over semi-quantitative scales, such as MASI, or scales used to assess color spaces, such as L*a*b.

After the treatments, the patient, together with a doctor or cosmetologist, assesses the effect obtained. The assessment is an individual decision of the person conducting a series of treatments, which does not provide an objective assessment of the effects of a given treatment therapy, and the number and severity of hyperpigmentation changes are determined in a unique way. In the diagnosis of skin discoloration, subjective and objective methods are used. The subjective scale is based on the visual assessment of excessively accumulated pigment in the skin and the assessment of changes in the size of a specific hyperpigmentation lesion. More accurate methods include objective methods such as clinical photography, ultrasonography and dermoscopy, and more precise quantitative methods such as hyper-spectral imaging and the GLCM method were used in this study to analyze and compare the treatment effects. Changes in the contrast values and homogeneity are shown.

This study showed that after microneedle mesotherapy with Vit. C improved skin lightening compared to kojic acid lightening, in which the contrast decreased and uniformity increased [36].

It should be noted that no current publications present the use of GLCM analysis in the quantitative assessment of hyperpigmentation lesions.

The study analyzed the photographic documentation of 12 volunteers with pigmented lesions on the forearm. The volunteers underwent a treatment of discoloration reduction using microneedle mesotherapy. Then, the photographic documentation of each of the volunteers participating in the study, before and after a series of four microneedle mesotherapy treatments, was analyzed. GLCM mathematical analysis was used to assess discoloration before the procedure and to eliminate it after the procedure. Contrast, uniformity and brightness of the treated skin were assessed by GLCM.

The GLCM contrast is a measure of the local variation in pixels, whereas the homogeneity provides information about the homogeneity of the pixels present in the image. This means that the greater the homogeneity and the lower the contrast, the more homogeneous the skin color is (no pigmentation changes). However, the greater the contrast and the lower the homogeneity, the more differentiated the skin tone and the more intense the pigmentation changes are [37].

Research conducted for the purposes of this study confirms the positive effect of microneedle mesotherapy on the reduction of discoloration. This research was carried out in order to confirm the hypothesis of the possibility of using the proposed GLCM analysis method to study the effectiveness of cosmetic procedures.

Due to the fact that this is a preliminary study and a relatively small study group, the statistical significance of the obtained differences has not been established. In subsequent studies, the authors plan to recruit a much larger research group. Nevertheless, the tendency where the results have the highest differences recorded in channel B is in line with the expectations and the initial research hypothesis.

However, the limitations of the proposed method should be noted. This limitation is the difficulty in comparing the results obtained using the same method of image analysis and processing (GLCM) but used for images with different input parameters. Even changing the resolution of the input image can affect the results. Therefore, when comparing the contrast and homogeneity of GLCM in studies conducted in different centers, the direction of research on the correlation between individual pixels and the resolution of the input image should be taken into account.

After the analysis of clinical photographs taken before and after a series of four microneedle mesotherapy treatments, the results obtained using the GLCM analysis showed a simultaneous decrease in GLCM contrast, an increase in GLCM homogeneity and an increase in brightness, which translates into even skin tone, as well as reducing the visibility of hyperpigmentation changes.

The use of gray-level comorbidity matrix analysis in assessing the effectiveness of individual therapies used in cosmetology has a chance of great success and will enable an objective comparison of treatment effects.

## 5. Conclusions

Microneedle mesotherapy in combination with vitamin C reduces hyperpigmentation changes in the skin, unifies the skin tone and reduces the visibility of hyperpigmentation changes. The use of GLCM analysis enables a quantitative, precise assessment of hyperpigmentation changes in a given area of the skin and can be widely used in cosmetology to assess therapeutic effects.

## Figures and Tables

**Table 1:** Brightness before and after microneedle mesotherapy treatment for individual patients.

BRIGHTNESS		
Patient	BEFORE Procedure	AFTER Procedure
1	165.25	196.35
2	163.25	175.36
3	125.36	142.36
4	184.36	199.36
5	165.52	175.54
6	175.21	187.52
7	136.98	154.23
8	169.36	180.23
9	152.52	185.25
10	145.55	165.32
11	139.25	174.32
12	150.63	159.37

**Table 2:** GLCM contrasts before and after needle mesotherapy treatment for individual patients.

GLCM CONTRAST		
Patient	BEFORE Procedure	AFTER Procedure
1	8.41	7.65
2	8.66	7.63
3	9.68	8.55
4	7.35	6.32
5	6.69	6.41
6	4.56	4.32
7	7.87	7.02
8	5.69	4.99
9	8.37	7.54
10	6.29	5.98
11	8.88	7.68
12	7.54	6.66

**Table 3:** GLCM homogeneity before and after microneedle mesotherapy for individual patients.

GLCM HOMOGENEITY		
Patient	BEFORE Procedure	AFTER Procedure
1	0.65	0.68
2	0.58	0.68
3	0.85	0.89
4	0.52	0.74
5	0.52	0.63
6	0.63	0.69
7	0.66	0.71
8	0.58	0.87
9	0.74	0.86
10	0.65	0.87
11	0.66	0.72
12	0.36	0.41

**Table 4:** Average values of GLCM contrast and homogeneity in the appropriate channels of the RGB scale before and after the needle mesotherapy treatment.

	GLCM Contrast before the Procedure	GLCM Contrast after the Procedure	Homogeneity before the Procedure	Homogeneity after the Procedure
CANAL R	11.38	10.45	0.64	0.72
CANAL G	11.84	10.76	0.64	0.64
CANAL B	12.79	9.97	0.59	0.69

## Data Availability

Not applicable.
